# Fertility Preservation in Young Adult Patients with Rectal Cancer: A Few Things to Consider

**DOI:** 10.1093/oncolo/oyac148

**Published:** 2022-08-13

**Authors:** Haiyang Zhou

**Affiliations:** Department of Colorectal Surgery, Changzheng Hospital, Naval Medical University, Shanghai, People’s Republic of China

## Abstract

This article evaluates the toxicity and feasibility of gemcitabine/nab-paclitaxel/S-1 chemotherapy on a 21-day cycle in patients with locally advanced or metastatic pancreatic ductal adenocarcinoma, reporting dose-limiting toxicity and maximum tolerated dose of S-1 in this regimen.

In a recent issue of *The Oncologist*, Stal and colleagues reported that more than half of young adult patients with rectal cancer did not receive fertility counseling, and only one fifth of patients banked sperm (males) or eggs/embryos (females) prior to their cancer therapy, and older age at diagnosis and/or greater quality of life were significantly associated with a higher likelihood of fertility discussions.^1^ The authors concluded that the young adult patients with rectal cancer were at risk of inadequate fertility counseling and care. Some important things regarding this topic should be considered.

First, this study included patients younger than age 50 years at diagnosis, which is supported by the ASCO guideline of discussing possible treatment-related infertility with cancer patients who are of reproductive age. This was quite reasonable. Although the traditional upper age boundary for young adult patients with rectal cancer is 39 years, it is important to also include individuals between ages 40 and 49 years when discussing fertility preservation, because of the current shift toward delaying family building and the rising age of parenthood.^2^ In fact, for male patients, no strict upper age boundary should be made, since the biological potential for reproduction continues for most of the men’s lives.

Second, the stage of the disease should be taken into consideration when discussing possible fertility preservation. For early-stage rectal cancer, a local resection to cure would be unlikely to affect fertility. For advanced-stage rectal cancer, fertility can be impaired to different extents, due to the single or combined modality therapies including radical surgery, cytotoxic chemotherapies, and pelvic radiation.^3^ Because of the progress in immune therapy, fertility preservation was achieved in a young adult patient with rectal cancer with mismatch repair gene deficiency (dMMR) or microsatellite instability (MSI) who got a complete response after neoadjuvant treatment with PD-1 blockage.^4^

Third, the options for fertility preserving approaches should be communicated by the clinical practitioner to ensure that the young adult patients with rectal cancer are adequately counseled and cared for. For male patients, sperm banking and egg harvesting should be routinely offered. For female patients, when there is insufficient time for performing ovarian stimulation with the purpose of obtaining oocytes or embryos for cryopreservation, ovarian tissue harvesting, and cryopreservation is a viable option, which is typically achieved via a minimally invasive procedure and does not require ovarian stimulation.^5^
